# Assessment of enhanced recovery after surgery protocol in older adults undergoing total knee arthroplasty

**DOI:** 10.1097/MD.0000000000048011

**Published:** 2026-03-27

**Authors:** Pengxi He, Qiang Xu, Yong Feng

**Affiliations:** a Department of Traumatic Orthopedics, Affiliated Hospital of Yan’an University, Yan’an City, Shaanxi Province, China; b Department of Trauma Repair Surgery, Affiliated Hospital of Yan’an University, Yan’an City, Shaanxi Province, China; c Department of Joint Surgery, Affiliated Hospital of Yan’an University, Yan’an City, Shaanxi Province, China.

**Keywords:** aged, enhanced recovery after surgery (ERAS), perioperative optimization, postoperative rehabilitation, total knee arthroplasty

## Abstract

This study aimed to examine the influence of enhanced recovery after surgery (ERAS) application on postoperative recovery dynamics and safety profiles in elderly total knee arthroplasty (TKA) patients. A retrospective cohort analysis was performed on 400 individuals aged 65 years or older who underwent primary unilateral TKA between January 2022 and December 2023. According to perioperative management strategies, participants were divided into an ERAS group (n = 200) managed with a standardized multimodal care pathway and a conventional group (n = 200) receiving routine treatment. Comparative evaluations included perioperative parameters such as time to first ambulation, hospital stay duration (length of stay), pain intensity measured by the visual analog scale, knee range of motion at discharge, postoperative complication incidence, and overall patient satisfaction. No significant differences were found in baseline demographic or comorbidity profiles between the 2 cohorts (*P* >.05). Compared with the conventional management pathway, ERAS implementation was associated with a significantly shorter length of stay (6.20 ± 1.80 vs 9.50 ± 2.30 days, *P* <.001), earlier mobilization (1.60 ± 0.50 vs 2.80 ± 0.90 days, *P* <.001), lower visual analog scale pain scores on postoperative days 1 and 3 (both *P* <.001), and greater knee flexion at discharge (105.30 ± 9.40° vs 92.60 ± 10.80°, *P* <.001). Although the overall complication rate was numerically lower in the ERAS group (6.0% vs 8.5%, *P* = .358), the difference was not statistically significant. Notably, patient satisfaction markedly improved in the ERAS cohort (92.5% vs 78.0%, *P* = .001). Implementation of the ERAS pathway in elderly patients undergoing TKA significantly enhances postoperative functional recovery, reduces pain, and increases satisfaction while maintaining a comparable safety profile. These findings support ERAS as a reliable, multidisciplinary, and cost-effective perioperative management strategy for geriatric TKA care. These findings suggest potential clinical and healthcare efficiency benefits; however, formal economic evaluations are required to confirm cost-effectiveness.

## 1. Introduction

Total knee arthroplasty (TKA) is the definitive surgical treatment for end-stage knee osteoarthritis, offering effective pain relief, deformity correction, and functional restoration.^[1]^ With the demographic shift toward an aging society, the proportion of elderly patients undergoing TKA continues to rise, creating new challenges for perioperative management.^[2]^ Older individuals often present with reduced physiological reserve, multiple comorbidities, and poorer baseline functional status, all of which can prolong hospitalization, slow rehabilitation, and increase the risk of adverse outcomes.^[3,4]^

The enhanced recovery after surgery (ERAS) concept was originally proposed by Kehlet and collaborators in the late 1990s as an evidence-based, multidisciplinary perioperative approach aimed at reducing surgical stress and facilitating early postoperative rehabilitation.^[5]^ In recent years, ERAS programs have been widely implemented across surgical disciplines and have consistently demonstrated reductions in-hospital length of stay (LOS), complication rates, and healthcare costs, alongside improvements in functional and patient-reported outcomes.^[6,7]^ Within the field of joint arthroplasty, growing evidence suggests that ERAS pathways can reduce LOS, alleviate early postoperative pain, and expedite functional recovery without increasing complication rates.^[8,9]^

Nevertheless, the application of ERAS principles in elderly TKA populations remains insufficiently explored. Physiological frailty, diminished organ reserve, and the prevalence of comorbidities may alter the recovery trajectory and affect ERAS tolerance.^[10–12]^ Moreover, data concerning safety endpoints – such as thromboembolic events and wound complications – are still limited.^[13]^ With the accelerating global trend of population aging, especially across Asian countries, there is a growing need for robust clinical evidence to further substantiate the safety and effectiveness of ERAS in elderly patients undergoing TKA. Accordingly, this retrospective cohort study included 400 elderly patients who underwent primary TKA, with 200 managed through an ERAS protocol and 200 treated using conventional perioperative care. The investigation evaluated the influence of ERAS on perioperative recovery parameters, postoperative pain management, knee function, incidence of complications, and overall patient satisfaction. The ultimate goal was to enhance perioperative strategies for geriatric TKA and to provide empirical support for the evidence-based application of ERAS within this high-risk population.

## 2. Methods

### 2.1. Study design and population

This study was approved by the Ethics Committee of Affiliated Hospital of Yan’an University. This retrospective analysis included elderly individuals who received primary TKA at our center during the period from January 2022 to December 2023. A total of 400 cases were included and assigned to 2 groups according to the perioperative management protocol: the ERAS group (n = 200), managed under a multimodal enhanced recovery program, and the conventional group (n = 200), treated with standard perioperative care. Ethical approval for this study was obtained from the institutional review board, and all experimental procedures were conducted in accordance with the ethical principles of the Declaration of Helsinki. Because of the retrospective design, no a priori sample size calculation was performed; however, the final cohort size was adequate for exploratory comparative analysis.

Inclusion criteria:

1. Individuals who were 65 years of age or older at the time of surgery.
2. Patients diagnosed with end-stage knee osteoarthritis and indicated for unilateral primary TKA.
3. Patients with comprehensive clinical documentation and complete follow-up data.

Exclusion criteria:

1. Revision or bilateral TKA;
2. Severe cardiopulmonary, hepatic, or renal dysfunction;
3. Preoperative infection or inflammatory arthritis;
4. Incomplete data or lost follow-up.

### 2.2. ERAS pathway and conventional management

Patients in the ERAS group received standardized perioperative management according to enhanced recovery principles, which included:

Preoperative education: individual counseling on procedure expectations, nutrition, and psychological readiness;

Optimized anesthesia: regional block combined with multimodal analgesia;

Early mobilization: bedside sitting and assisted ambulation within 24 hours after surgery;

Early oral feeding: initiation of fluids within 6 hours postoperatively;

Thrombosis prevention: mechanical compression and low-molecular-weight heparin prophylaxis;

Standardized rehabilitation: progressive knee flexion and muscle strengthening guided by physiotherapists.

The conventional group received traditional perioperative management, including fasting before surgery, delayed postoperative feeding (≥24 hours), longer immobilization (≥48 hours), and analgesia primarily based on opioid administration.

### 2.3. Surgical procedure

All operations were performed by the same orthopedic team using a medial parapatellar approach. A standard posterior-stabilized prosthesis was implanted in all cases. Tourniquet use, bone cement fixation, and drainage management followed routine protocols. Postoperatively, antibiotics were administered for 24 hours for infection prevention, and thromboembolic prophylaxis was implemented according to patient risk level.

### 2.4. Observation indicators

Perioperative and postoperative clinical indicators were recorded and analyzed, including: baseline characteristics: age, sex, BMI, ASA grade, diabetes, and hypertension; perioperative outcomes: time to first ambulation, length of hospital stay, pain score (visual analog scale) on postoperative day 1 and day 3, and range of motion (ROM) at discharge; complications: including deep vein thrombosis, wound infection, and nausea/vomiting; patient satisfaction: measured at the time of discharge through a 5-level Likert scale ranging from “very dissatisfied” to “very satisfied.” Patients rating their experience as either “satisfied” or “very satisfied” were considered satisfied.

### 2.5. Statistical analysis

All data analyses were carried out using SPSS software, version 26.0 (IBM Corp., Armonk). Quantitative variables were summarized as mean ± standard deviation (x̄ ± s) and compared using independent-sample *t*-tests, whereas qualitative variables were expressed as counts and proportions (n [%]) and examined using the χ^2^ test or Fisher exact test when applicable. Statistical significance was defined as a 2-sided *P*-value <.05.

## 3. Results

### 3.1. Baseline characteristics

A total of 400 elderly individuals who received TKA were analyzed, comprising 200 patients treated following the ERAS protocol and 200 managed with traditional perioperative care. Baseline demographic and clinical parameters – including age, gender distribution, BMI, ASA status, and common comorbidities (e.g., diabetes and hypertension) – showed no significant intergroup differences (*P* >.05), confirming the comparability of the 2 populations (Table 1).

**Table 1:** Comparison of baseline characteristics between the 2 groups.

Variable	ERAS group (n = 200)	Conventional group (n = 200)	χ^2^/ *t*-value	*P*-value
Age (yr, mean ± SD)	72.80 ± 5.90	73.10 ± 6.10	*t* = 0.484	.628
Female n (%)	136 (68.00%)	132 (66.00%)	χ^2^ = 0.167	.685
BMI (kg/m^2^)	26.40 ± 3.50	26.10 ± 3.80	*t* = 0.814	.417
ASA III–IV n (%)	78 (39.00%)	83 (41.50%)	χ^2^ = 0.209	.647
Diabetes n (%)	56 (28.00%)	61 (30.50%)	χ^2^ = 0.259	.611
Hypertension n (%)	103 (51.50%)	108 (54.00%)	χ^2^ = 0.228	.633

Continuous variables are presented as mean ± standard deviation, and categorical variables as numbers with corresponding percentages (n [%]).

ASA = American Society of Anesthesiologists, BMI = body mass index.

### 3.2. Perioperative outcomes

**Figure 1.**
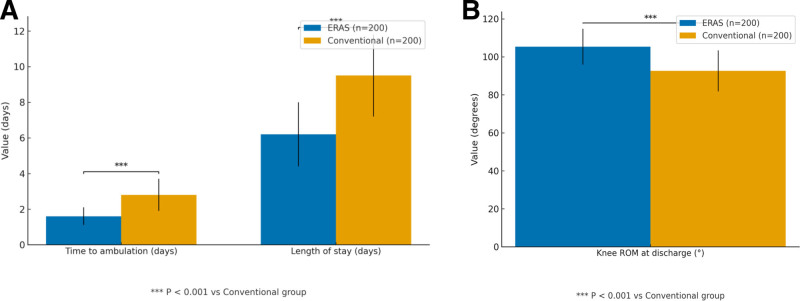
(A) Early recovery parameters in patients managed with ERAS versus conventional perioperative care. (B) Knee range of motion at discharge in patients managed under ERAS and conventional perioperative care. ERAS = enhanced recovery after surgery.

**Figure 2.**
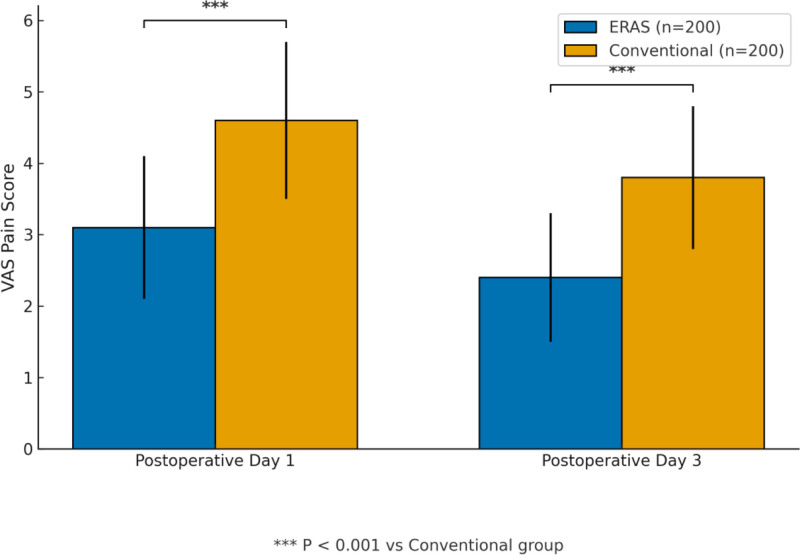
Comparison of postoperative pain scores (VAS) between ERAS and conventional groups. ERAS = enhanced recovery after surgery, VAS = visual analogue scale.

Compared with conventional care, patients managed under the ERAS protocol showed significantly accelerated postoperative recovery, characterized by earlier ambulation (1.60 ± 0.50 vs 2.80 ± 0.90 days; *t* = 16.783, *P* <.001), reduced length of hospital stay (6.20 ± 1.80 vs 9.50 ± 2.30 days; *t* = 17.234, *P* <.001), improved knee ROM at discharge (105.30 ± 9.40° vs 92.60 ± 10.80°; *t* = 12.702, *P* <.001), and lower visual analog scale pain scores on postoperative days 1 and 3 (all *P* <.001). A summary of these results is presented in Table 2 and Figs. 1A, B, and 2.

**Table 2:** Comparison of perioperative outcomes between the 2 groups.

Indicator	ERAS group	Conventional group	*t*-value	*P*-value
Hospital stay (d)	6.20 ± 1.80	9.50 ± 2.30	17.234	<.001
Time to ambulation (d)	1.60 ± 0.50	2.80 ± 0.90	16.783	<.001
VAS day 1	3.10 ± 1.20	4.60 ± 1.50	13.095	<.001
VAS day 3	2.40 ± 1.00	3.80 ± 1.30	14.071	<.001
Knee ROM at discharge (°)	105.30 ± 9.40	92.60 ± 10.80	12.702	<.001

Values are presented as mean ± standard deviation.

ERAS = enhanced recovery after surgery, ROM = range of motion, VAS = visual analogue scale.

** *P* < .001 compared with the conventional group.

### 3.3. Postoperative complications

Postoperative complications occurred less frequently in patients managed under ERAS (6.0%) compared with those receiving conventional care (8.5%), although this difference did not reach statistical significance (χ^2^ = 0.847, *P* = .358). Patients managed under the ERAS protocol experienced marginally reduced rates of deep vein thrombosis, wound infection, and postoperative nausea/vomiting relative to the conventional care group (Table 3). No in-hospital deaths were reported, indicating that ERAS implementation did not elevate perioperative risk.

**Table 3:** Comparison of postoperative complications between the 2 groups.

Complication	ERAS group	Conventional group	χ^2^ value	*P*-value
Deep vein thrombosis	6 (3.00%)	8 (4.00%)	0.296	.585
Wound infection	3 (1.50%)	5 (2.50%)	0.523	.470
Nausea/vomiting	5 (2.50%)	9 (4.50%)	1.172	.279
Overall complications	12 (6.00%)	17 (8.50%)	0.847	.358

ERAS = enhanced recovery after surgery.

### 3.4. Patient satisfaction

A significantly greater proportion of patients in the ERAS group reported satisfaction with their care (92.5%) compared with the conventional group (78.0%) (χ^2^ = 10.835, *P* = .001). This difference indicates that ERAS implementation contributed to improved patient-perceived care quality and overall postoperative recovery experience (Table 4).

**Table 4:** Comparison of postoperative patient satisfaction between the 2 groups.

Satisfaction level	ERAS group	Conventional group	χ^2^ value	*P*-value
Satisfied/very satisfied	185 (92.50%)	156 (78.00%)	10.835	.001

ERAS = enhanced recovery after surgery.

## 4. Discussion

This study suggests that in elderly patients undergoing primary TKA, implementation of an ERAS pathway significantly enhanced early mobilization, reduced LOS, relieved pain, improved knee ROM at discharge, and increased patient satisfaction – without any rise in perioperative complications. These findings highlight both the efficacy and safety of ERAS in geriatric arthroplasty practice.

Our observations are consistent with prior meta-analyses reporting that ERAS shortens LOS, reduces postoperative pain, and accelerates functional recovery.^[14,15]^ For example, a comprehensive systematic review of 47 studies encompassing approximately 76 000 arthroplasty cases showed that ERAS reduced LOS (WMD = –2.65 days) and improved ROM (WMD = +6.65°) without increasing readmission or mortality. The present study extends these benefits specifically to elderly TKA, confirming comparable magnitudes of improvement. Early mobilization within 24 hours – a core ERAS component – has been independently associated with shorter LOS and fewer complications among older TKA patients.^[16]^

The underlying mechanisms likely involve attenuation of surgical stress, optimized analgesia, and earlier functional loading.^[17,18]^ Multimodal analgesia effectively reduces opioid consumption and related adverse effects – such as nausea, vomiting, and postoperative delirium – that are particularly problematic in geriatric populations.^[19]^ Early ambulation prevents venous stasis, preserves muscle strength, and may reduce thromboembolic risk, thereby supporting safer recovery in the elderly.^[20]^ The comparable complication rates observed between ERAS and conventional groups further suggest that, with careful patient selection and standardized multidisciplinary implementation, ERAS does not compromise safety.

From a clinical perspective, these outcomes are highly relevant. As population aging increases surgical demand, streamlined perioperative pathways that enable faster recovery and shorter hospitalization have major implications for healthcare efficiency and quality of care. The notably high satisfaction rate observed in the ERAS group (92.5%) underscores the acceptability of this approach among elderly TKA patients. Importantly, our findings indicate that advanced age alone should not preclude ERAS application when supported by a comprehensive, team-based model of care.

Despite the encouraging findings of this study, several limitations should be acknowledged to ensure a balanced interpretation. Although the ERAS pathway appeared to reduce hospital LOS and resource utilization, this study did not include a formal health economic analysis; therefore, our previous description of the intervention as “cost-effective” may be overstated. While shorter hospitalization and accelerated recovery suggest potential economic advantages, future prospective studies incorporating formal cost analyses are necessary to confirm the true economic impact of ERAS implementation in elderly TKA populations. In addition, the retrospective design inherently introduces the possibility of selection bias, as patient allocation to ERAS or conventional care was based on perioperative management decisions rather than randomized assignment, potentially influenced by patient characteristics, surgeon preference, or temporal practice changes; thus, residual confounding cannot be entirely excluded despite comparable baseline characteristics. The relatively low complication rates observed in both groups may also limit statistical power to detect modest differences in adverse events, meaning that larger multicenter cohorts or prospective trials are required to more definitively evaluate safety outcomes. Furthermore, this single-center study, conducted with standardized surgical teams and perioperative protocols, enhances internal consistency but may restrict generalizability to other healthcare settings with different patient populations, institutional resources, or ERAS implementation strategies. Long-term functional outcomes were not assessed, and factors such as sustained knee function, implant survivorship, quality of life, and late complications remain important considerations that warrant future longitudinal evaluation. Finally, subjective outcomes such as patient satisfaction may be susceptible to performance bias, particularly in enhanced recovery programs where enhanced education, communication, and clinical attention may positively influence patient perception independently of actual clinical recovery. Taken together, these limitations highlight the need for well-designed prospective multicenter studies with long-term follow-up and formal economic evaluation to further validate the benefits of ERAS protocols in geriatric TKA care.

## 5. Conclusion

Adoption of an ERAS pathway for elderly individuals receiving TKA significantly facilitated early mobilization, minimized hospital stay and postoperative discomfort, improved functional recovery, and enhanced patient satisfaction, with no observed rise in complication rates. Collectively, these findings support ERAS as a reliable, safe, and multidisciplinary perioperative care model that merits broader clinical implementation in geriatric TKA.

## Author contributions

**Conceptualization:** Pengxi He, Qiang Xu, Yong Feng.

**Data curation:** Pengxi He, Qiang Xu, Yong Feng.

**Formal analysis:** Pengxi He, Qiang Xu, Yong Feng.

**Funding acquisition:** Pengxi He, Qiang Xu, Yong Feng.

**Investigation:** Yong Feng.

**Writing** – **original draft:** Pengxi He, Yong Feng.

**Writing** – **review & editing:** Pengxi He, Yong Feng.
